# Inappropriate Ventilatory Homeostatic Responses in Hospitalized COVID-19 Patients

**DOI:** 10.3389/fneur.2022.909915

**Published:** 2022-06-15

**Authors:** Prem Jareonsettasin, Claudia Zeicu, Beate Diehl, Ronald M. Harper, Rónan Astin

**Affiliations:** ^1^Department of Clinical and Experimental Epilepsy, Queen Square Institute of Neurology, University College London, London, United Kingdom; ^2^Division of Medical Specialties, University College London Hospitals NHS Foundation Trust, London, United Kingdom; ^3^Department of Clinical Neurophysiology, University College London Hospitals NHS Foundation Trust National Hospital for Neurology and Neurosurgery, London, United Kingdom; ^4^Department of Neurobiology and the Brain Research Institute, David Geffen School of Medicine, University of California, Los Angeles, Los Angeles, CA, United States; ^5^NIHR University College London Hospitals Biomedical Research Centre, London, United Kingdom

**Keywords:** ventilation, impaired homeostasis, COVID-19, breathing pattern disorder, dyspnea, post-covid breathing pattern dysfunction

## Abstract

**Background:**

The clinical presentation of COVID-19 suggests altered breathing control - tachypnoea, relative lack of dyspnoea, and often a discrepancy between severity of clinical and radiological findings. Few studies characterize and analyse the contribution of breathing drivers and their ventilatory and perceptual responses.

**Aim:**

To establish the prevalence of inappropriate ventilatory and perceptual response in COVID-19, by characterizing the relationships between respiratory rate (RR), dyspnoea and arterial blood gas (ABG) in a cohort of COVID-19 patients at presentation to hospital, and their post-Covid respiratory sequelae at follow-up.

**Methods:**

We conducted a retrospective cohort study including consecutive adult patients admitted to hospital with confirmed COVID-19 between 1st March 2020 and 30th April 2020. In those with concurrent ABG, RR and documented dyspnoea status on presentation, we documented patient characteristics, disease severity, and outcomes at hospital and 6-week post-discharge.

**Results:**

Of 492 admissions, 194 patients met the inclusion criteria. Tachypnoea was present in 75% pronounced (RR>30) in 36%, and persisted during sleep. RR correlated with heart rate (HR) (r = 0.2674), temperature (r = 0.2824), CRP (r = 0.2561), Alveolar-arterial (A-a) gradient (r = 0.4189), and lower PaO_2_/FiO_2_ (PF) ratio (r = −0.3636). RR was not correlated with any neurological symptoms. Dyspnoea was correlated with RR (r = 0.2932), A-a gradient (r = 0.1723), and lower PF ratio (r = −0.1914), but not correlated with PaO_2_ (r = −0.1095), PaCO_2_ (r = −0.0598) or any recorded neurological symptom except for altered consciousness. Impaired ventilatory homeostatic control of pH/PaCO_2_ [tachypnoea (RR>20), hypocapnia (PaCO_2_ <4.6 kPa), and alkalosis (pH>7.45)] was observed in 29%. This group, of which 37% reported no dyspnoea, had more severe respiratory disease (A-a gradient 38.9 vs. 12.4 mmHg; PF ratio 120 vs. 238), and higher prevalence of anosmia (21 vs. 15%), dysgeusia (25 vs. 12%), headache (33 vs. 23%) and nausea (33 vs. 14%) with similar rates of new anxiety/depression (26 vs. 23%), but lower incidence of past neurological or psychiatric diagnoses (5 vs. 21%) compared to appropriate responders. Only 5% had hypoxia sufficiently severe to drive breathing (i.e. PaO_2_ <6.6 kPa). At 6 weeks post-discharge, 24% (8/34) showed a new breathing pattern disorder with no other neurological findings, nor previous respiratory, neurological, or psychiatric disorder diagnoses.

**Conclusions:**

Impaired homeostatic control of ventilation i.e., tachypnoea, despite hypocapnia to the point of alkalosis appears prevalent in patients admitted to hospital with COVID-19, a finding typically accompanying more severe disease. Tachypnoea prevalence was between 12 and 29%. Data suggest that excessive tachypnoea is driven by both peripheral and central mechanisms, but not hypoxia. Over a third of patients with impaired homeostatic ventilatory control did not experience dyspnoea despite tachypnoea. A subset of followed-up patients developed post-covid breathing pattern disorder.

## Introduction

Early descriptive studies of COVID-19 clinical presentations found tachypnoea, a relative lack of dyspnoea, and often a discrepancy between severity of respiratory clinical signs and radiological findings. Some have described the combination of phenomena as “silent” or “happy” hypoxemia (1–4), inferring dysfunctional regulatory breathing mechanisms.

However, data on the nature of breathing control in COVID-19 are lacking. A few previous studies investigated blood gas analysis in hospitalized COVID-19 patients (5–8), but none concurrently assessed arterial blood gases (ABG), respiratory rate and perception of dyspnoea, and therefore could not directly comment on appropriateness of physiological and breathing perception responses, which we attempt to do here.

Various physiological mechanisms control breathing. Hypercapnia/acidosis drives automatic breathing in a negative feedback loop, while hypoxia only drives breathing when severe (i.e., PaO_2_ <6.6 kPa) (9–11). Additional drives include thermal, emotional, somatosensory, pulmonary afferents, wakefulness-related signals, and conscious volition. Their neural substrates span all levels of the neuraxis, from the periphery to rostral brain areas, overlapping many areas that process smell, taste, emotion, and arousal.

Neurological symptoms, predominantly anosmia, dysgeusia and altered mental status, occur in many COVID-19 patients (12). Accumulating evidence points toward infection of vascular and immune cells, but not CNS neurons, particularly not those of the brainstem and cerebellum (13) – areas where major breathing control sites lie, raising the possibility of altered breathing control during COVID-19 infection due to direct injury to ancillary support areas by the virus.

In this study, our aim was to establish the prevalence of inappropriate ventilatory and perceptual responses in COVID-19, by characterizing breathing responses during acute infection through investigating the relationship between respiratory rate (RR), dyspnoea and ABG in COVID-19 patients at presentation to hospital, their relationship to neurological symptoms and autonomic control dysfunction, and their post-Covid respiratory sequelae at follow-up.

## Methods

We retrospectively collected data from electronic medical records (EPIC, Milky Way, Verona, WI, USA) of consecutive patients with a nasopharyngeal PCR-positive COVID-19 diagnosis who presented to the Emergency Department (ED) of University College Hospital, London between 1st March 2020 and 30th April 2020. Excluded patients were those transferred from another hospital, those without ABG results within 4 h of presentation, undocumented dyspnoea status, and patients who were immediately intubated on arrival. All patients had a respiratory presentation of COVID-19 as their main reason for admission. There were no secondary diagnoses, but co-morbidities are listed in **Table 2**. Clinical data, including RR, HR and ABGs were part of the ED initial evaluation and used for analysis. Dyspnoea status was assigned as positive if any of the following were documented by the clerking clinician: dyspnoea, breathlessness, shortness of breath, air hunger, respiratory discomfort or respiratory distress. Arterial blood samples were processed on ABL90 FLEX gas analysers (Radiometer, Crawley, UK).

Physiological breathing response was considered inappropriate (“Inappropriate Responders”) in those who were simultaneously tachypnoeic (RR>20), hypocapnic (PaCO2 <4.6 kPa) and alkalotic (pH>7.45) (15). Otherwise, the response was considered appropriate (“Appropriate Responders”). No patients had RR <12, which would suggest a deficient respiratory response. Hypoxia was defined as PaO_2_ <10 kPa. Hypoxia sufficient to stimulate respiratory drive was defined as PaO_2_ <6.6 kPa. The breathing pattern assessment tool (BPAT) provides a validated score used to grade the severity and make the diagnosis of breathing pattern disorder (BPD), with a BPAT score of 4 or more corresponding to a diagnosis of BPD (16). BPD was assessed using the BPAT for patients who attended face-to-face follow-up appointments.

Correlations between continuous variables were evaluated using Pearson's correlation and described using median and inter-quartile range (IQR); mortality rates between groups were compared using the χ2 test. Data were analyzed using Prism 8 (GraphPad, San Diego, CA, USA). The study was approved by the Westminster Research Ethics Committee (NHS Health Research Authority, IRAS no: 284088).

## Results

Of 492 patients admitted with COVID-19 during the study period, 194 had concurrent ABG, RR and documented dyspnoea status, and were therefore included in the study.

**Figure 1:**
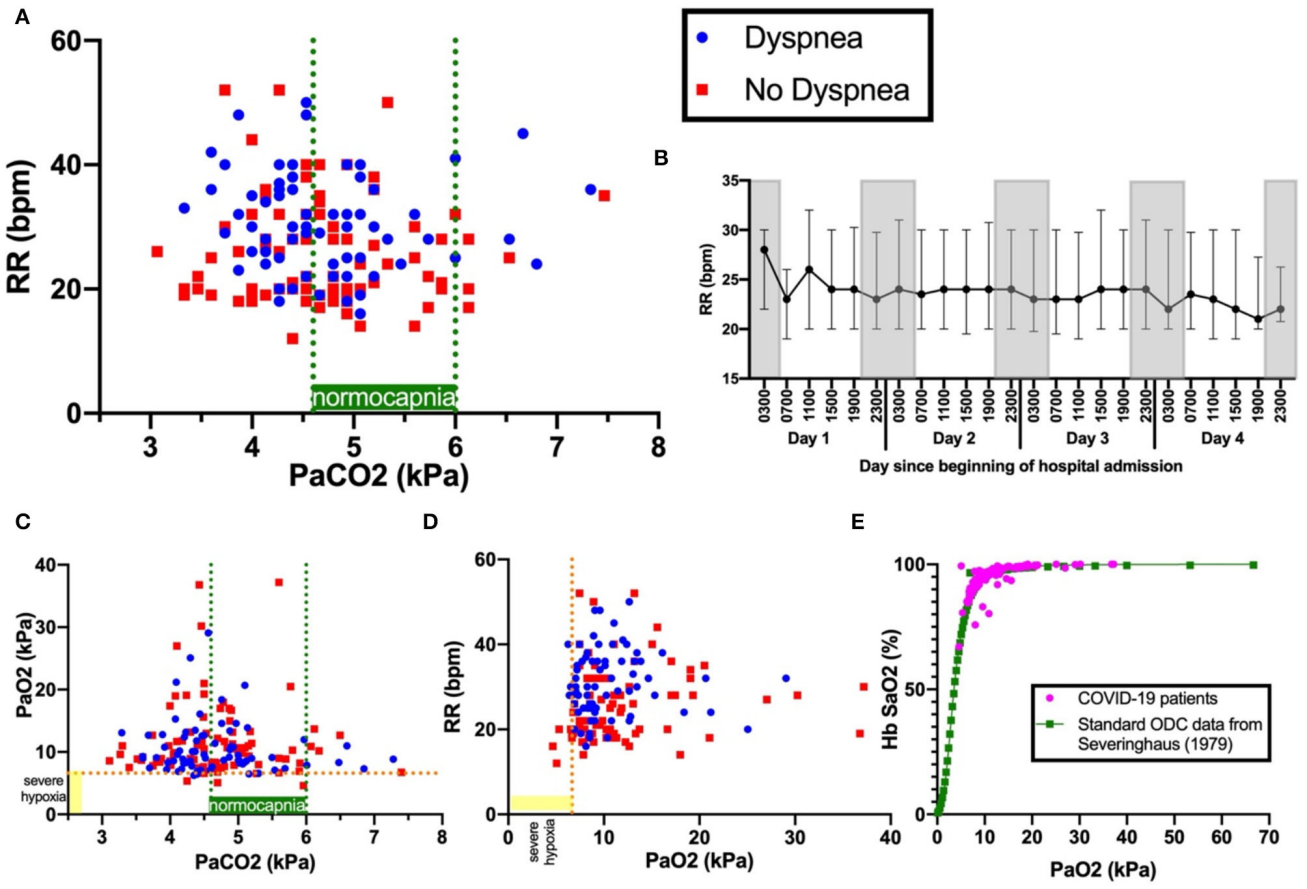
Panel **(A)** shows PaCO2 versus RR. Blue dots indicate patients with dyspnoea; red squares, patients with no dyspnoea. Dashed green lines indicate boundaries of normocapnia (4.6kPa and 6.0kPa). Dashed orange line indicates severe hypoxia at 6.6kPa, a level sufficient to drive ventilation. Panel **(B)** shows the 4-hourly RR (median and interquartile range) over the first 96 hours since admission. Gray-shaded boxes indicate night. Panels **(C)** show PaCO_2_ versus PaO_2_ and **(D)**PaCO_2_ versus RR. Panel **(E)** shows the oxygen dissociation curve (ODC) of Covid-19 patients in this cohort plotted against standard human ODC data from (14), showing no shift.

Tachypnoea was common and pronounced: 75% (146/194) exhibited RR>20 breaths per minute (bpm), RR>25 bpm in 57% (69/194), RR>30 bpm in 36% (42/194), RR>35 bpm in 22% (42/194), and RR>40 bpm in 8%(16/194) (Figure 1A). RR at 1 and 2 h following arterial blood sampling varied little (average standard deviation: 2.8 bpm). Notably, tachypnoea was maintained with little variation both day and night over the 96 h following admission, which included sleep periods (Figure 1B). No patients in our cohort had RR <12, which would indicate depressed respiratory drive.

RR correlated with HR (r = 0.267, *p* < 0.001), temperature (r = 0.282, *p* < 0.001), CRP (r = 0.256, *p* < 0.001), A-a gradient (r = 0.419, *p* < 0.001), and inversely correlated with PF ratio (r = −0.363, *p* < 0.001). RR did not correlate with mortality (r = 0.0319, *p* = 0.692), mean arterial pressure (MAP) (r-0.113, *p* = 0.115), anosmia, dysgeusia, headache, dizziness, altered consciousness, nausea, seizure, new anxiety or depression (Table 1). There was no shift in the oxygen dissociation curve in our cohort (Figure 1E).

**Table 1:** Relationship between neurological, autonomic, biochemical variables and RR and dyspnea.

	**RR (Pearson's correlation)**		**Dyspnea status**		
	**r-value**	***p*** **-value**	**Dyspnea (*n* = 86) (%)**	**No Dyspnea (*n* = 108) (%)**	***p*** **-value (Chi-square)**
**Neurological symptoms**					
Anosmia	0.055	0.4446	15 (17)	12 (11)	0.2057
Dysgeusia	0.041	0.5735	18 (21)	13 (12)	0.0878
Headache	−0.009	0.9025	24 (28)	27 (25)	0.6477
Dizziness	−0.058	0.4238	3 (3)	7 (6)	0.3489
Nausea	0.025	0.7270	20 (23)	20 (19)	0.4178
Altered consciousness	−0.114	0.1128	3 (3)	25 (23)	**0.0001**
Seizure	0.109	0.1309	1 (1)	2 (2)	0.6992
Psychiatric (New anxiety or new depression)	−0.043	0.5510	21 (24)	26 (24)	0.9556
	**RR (Pearson's correlation)**		**Dysnea (Pearson's correlation)**		
	**r-value**	***p*** **-value**	**r-value**		***p*** **-value**
**Autonomic variables**				
HR	0.2674	**0.0002**	0.1300		0.0708
MAP	0.1134	0.1153	0.1319		0.0668
Temperature	0.2824	**<0.0001**	0.1673		**0.0198**
RR	n/a	n/a	0.2932		**<0.0001**
**Respiratory variables**				
pH	−0.0568	0.4312	0.0988		0.1703
PaCO2	−0.1242	0.0844	−0.0598		0.4078
PaO2	−0.1142	0.1128	−0.1095		0.1284
PF	−0.3636	**<0.0001**	−0.1914		**0.0075**
A-a gradient	0.4189	**<0.0001**	0.1723		**0.0163**
**Laboratory variables**				
Hb	0.0838	0.2489	0.1492		**0.0395**
Lym	0.1316	0.088	−0.0918		0.2353
CRP	0.2561	**0.0008**	0.1232		0.1106
D-dimer	0.1556	0.1111	0.1979		**0.042**
LDH	0.1688	0.0666	0.1668		0.0698
Ferritin	−0.0172	0.8471	0.0208		0.8154
**Mortality**	0.0319	0.692	−0.0412		0.5686

*Bold values meant statistically significant*.

Dyspnoea was present in 44% (86/194) of all patients, 48% (48/101) of those with hypoxia (PaO_2_ < 10 kPa), but only 33% (4/12) of those with hypoxia sufficient to stimulate respiratory drive (PaO_2_ < 6.7kPa) (Figures 1C,D). Dyspnoea was weakly correlated with RR (r = 0.293, *p* < 0.001), temperature (r = 0.167, *p* = 0.020), A-a gradient (r = 0.172, *p* = 0.016), Hb (r = 0.149, *p* = 0.040) and D-dimer (r = 0.198, *p* = 0.042). Dyspnoea was not correlated with pH (r = 0.099, *p*-0.170), PaCO_2_ (*r* = −0.060, *p* = 0.408) or PaO_2_ (r = −0.110, *p* = 0.128), nor mortality (r = −0.041, *p* = 0.569). Dyspnoea was weakly inversely correlated to PF ratio (r = −0.191, *p* = 0.008). Except for altered consciousness (p=0.002), dyspnoea was not correlated with other neurological symptoms.

Inappropriate responders accounted for 29% (57/194). Respiratory alkalosis was associated with more severe respiratory disease; higher FiO_2_ (0.60 vs. 0.32, *p* < 0.001), greater A-a gradient (38.9 vs. 12.4 mmHg, *p* = 0.002), and lower PF ratio (120 vs. 238, *p* = 0.002).Markers of inflammation were also higher in this group [LDH (498 vs. 386 IU/L, *p* < 0.001), Ferritin (1,430 vs. 948 ug/L, *p* = 0.018)], but no significant difference in mortality was observed (30 vs. 40%, *p* = 0.195). The prevalence of severe hypoxia was low and similar in both groups (5 vs. 4%). However, inappropriate responders had higher rates of supplemental oxygen use (84 vs. 66%) indicating a higher level of underlying pre-hospital hypoxia that was immediately corrected on admission. Anosmia (21 vs. 11%), dysgeusia (25 vs. 12%), headache (33 vs. 23%), nausea (33 vs. 14%) were more prevalent in inappropriate responders. There were no differences in new anxiety or depression (26 vs. 23%), and past neurological or psychiatric diagnoses were less prevalent (5 vs. 21%) in inappropriate responders. The two groups did not differ significantly in age, sex, BMI, ethnicity, cardiovascular and respiratory co-morbidities. Though inappropriate responders had higher rates of dyspnoea (63 vs. 36%), 37% did not report dyspnoea (Table 2).

**Table 2:** Characteristics of appropriate vs inappropriate responders.

**Characteristics**	**Appropriate Responders**	**Inappropriate Responders (Tacyhpnoeic RR>20**,	***p*** **-value**
	**[*n* = 137]**	**hypocapnic PaCO2 <4.6kPa alkalotic pH>7.45) [*n* = 57]**	
**Age (median + IQR)**	68 (51–80)	63 (50–74)	0.0708
**Female (%)**	47 (34)	17 (30)	0.6166
**BMI (median + IQR)**	26.7 (22.8–30.6)	28.0 (25.4–31.6)	0.0682
**Ethnicity (%)**			
White	73 (53)	33 (58)	0.6355
Black	19 (14)	10 (18)	0.5137
Asian	26 (19)	6 (11)	0.2025
Other Ethnic Background	7 (4)	6 (3)	0.2082
Unknown	12 (9)	2 (4)	0.2401
**Co-morbidities (%)**			
Cardiovascular Disorders	85 (62)	41 (72)	0.2475
Respiratory Disorders	43 (31)	12 (21)	0.1648
Asthma	22 (16)	5 (9)	
COPD	14 (10)	2 (4)	
ILD	2 (1)	2 (4)	
OSA	2 (2)	4 (1)	
Other respiratory disorders	2 (1)	1 (2)	
Neurological/Psychiatric Disorders	29 (21)	3 (5)	**0.0055**
Other co-morbidities	59 (43)	14 (25)	**0.0222**
**Neurological/Psychiatric Symptoms (%)**			
Anosmia	15 (11)	12 (21)	0.0719
Dysgeusia	17 (12)	14 (25)	0.0514
Headache	32 (23)	19 (33)	0.1567
Dizziness	6 (4)	4 (7)	0.4835
Nausea	21 (14)	19 (33)	**0.0065**
Altered consciousness	24 (18)	4 (7)	0.0726
Seizure	3 (2)	0 (0)	0.5568
New Anxiety or Depression	32 (23)	15 (26)	0.7140
Any neurological or psychiatric symptom	92 (67)	41 (72)	0.6113
**Respiratory Characteristics (median + IQR)**			
Respiratory rate (bpm)	25 (20–32)	30 (26–36)	**<0.0001**
pH	7.44 (7.40–7.62)	7.49 (7.48–7.41)	**<0.0001**
PaCO2 (kPa)	4.87 (4.40–5.29)	4.10 (3.78–4.36)	**<0.0001**
PaO2 (kPa)	10.10 (8.18–12.85)	9.02 (7.76–12.20)	0.2711
BE (mEq/L)	1.10 (−2.85–4.55)	0.70 (−1.00–2.25)	0.3848
FiO2	0.32 (0.21–0.60)	0.60 (0.32–0.90)	**0.0011**
Supplemental Oxygen (%)	90 (66)	48 (84)	**0.0094**
A-a gradient (mmHg)	12.4 (5.3–44.5)	38.9 (12.3–73.0)	**0.0001**
PF ratio	238 (134–328)	120 (74–276)	**0.0019**
Dyspnoea (%)	50 (36%)	36 (63)	**0.0008**
Severely hypoxemic (PaO2 <6.6kPa) (%)	6 (4)	3 (5)	0.7237
**CXR severity**			
Mild (%)	34 (25)	14 (25)	1.0000
Moderate (%)	29 (21)	22 (39)	**0.0192**
Severe (%)	31 (23)	16 (28)	0.4634
Unknown (%)	43 (31)	5 (9)	**0.0008**
**Other Clinical Observations (median + IQR)**			
Heart Rate (bpm)	93 (78–105)	102 (86–115)	**0.0048**
Mean Arterial Pressure (mmHg)	90 (78–104)	94 (86–102)	0.1547
Temperature (°C)	37.2 (36.6–38.0)	37.8 (37.2–38.7)	**0.0006**
**Admission Bloods (median + IQR)**			
Hb (g/L)	129 (110–140)	134 (123–144)	0.0733
Lym (×10∧9/L)	1.03 (0.64–1.46)	0.96 (0.73–1.39)	0.9053
CRP (mg/L)	98 (42–192)	102 (66–238)	0.1299
D-dimer (mg/L)	1.68 (0.69–2.94)	1.28 (0.69–4.0)	0.721
Troponin T (ng/L)	22 (10–45)	16 (10–23)	0.596
LDH (IU/L)	386 (295–510)	498 (391–600)	**0.0007**
Ferritin (ug/L)	948 (406–1,814)	1,430 (793–2,491)	**0.018**
**Mortality (%)**	55 (40)	17 (30)	0.1948

*Bold values meant statistically significant*.

**Figure 2:**
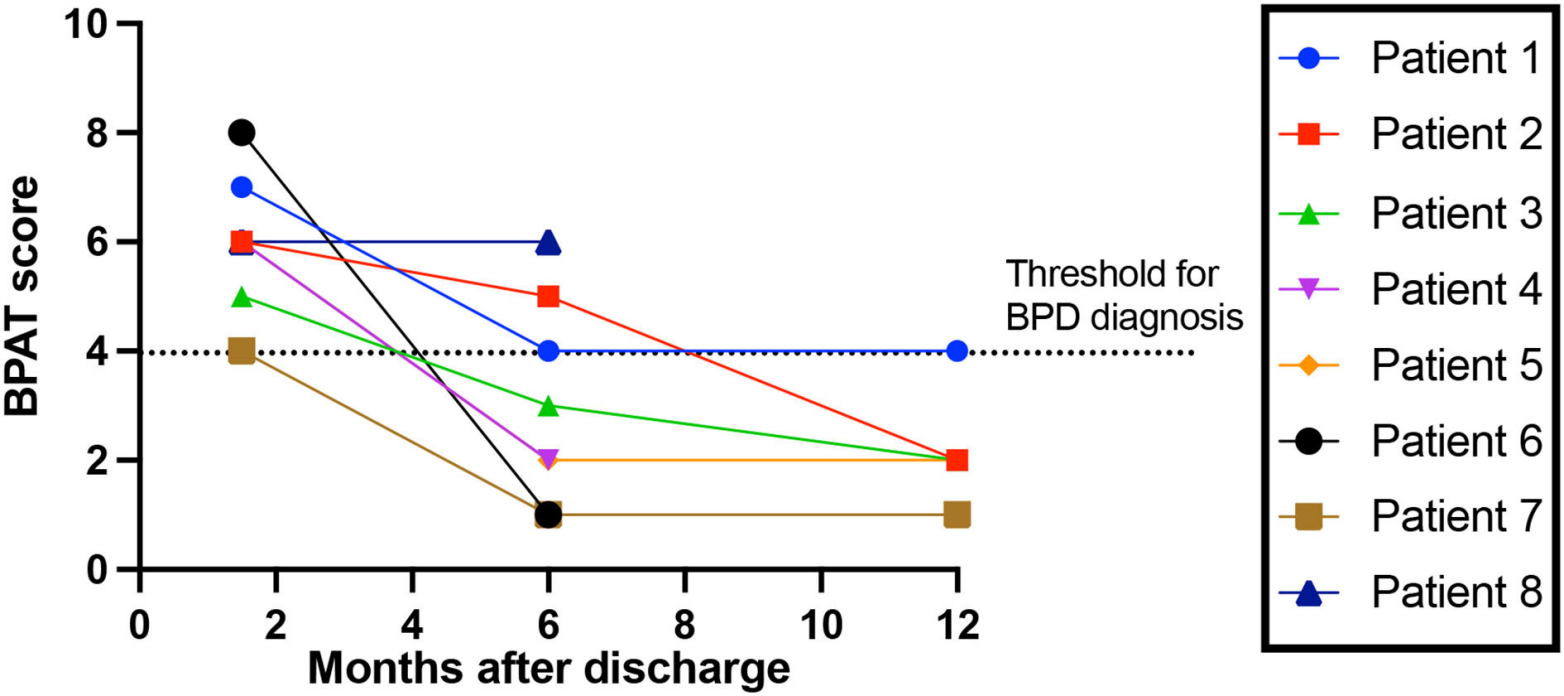
Breathing Pattern Disorder severity over time. Breathing Pattern Assessment Tool score to rate breathing pattern disorder (BPD) severity, over time since discharge from hospital. Threshold for BPD diagnosis is a score of 4 or more (16).

Of the total cohort, 38% (74/194), had a 6-week follow-up after hospital discharge, of which 34 (17.5%) were face-to-face consultations which allowed full clinical assessment. A new diagnosis of breathing pattern disorder (BPD) was made in 23.5% (8/34), with a BPAT score of 6 (5–7). None had a past medical history of respiratory, neurological or psychiatric disorder. None had other focal neurological findings on examination. 62.5% (5/8) were in the inappropriate responders group. All eight patients with BPD had 6-month follow-up, of which three still had BPD, with a BPAT score of 2.5 (3–5). Five of the eight patients attended 12-month follow-up, of which one had BPD, with a BPAT score of 2 (2, 3) (Figure 2).

## Discussion

Nearly a third of the patients in this study demonstrated impaired homeostatic control of ventilation i.e., tachypnoea, despite hypocapnia to the point of alkalosis, a finding accompanying more severe disease (as evidenced by worse physiological (higher FiO_2_, greater A-a gradient, and lower PF ratio) and inflammatory markers). Over a third showed a reduced dyspnoeic response to tachypnoea.

The prevalence of inappropriate responders is consistent with estimates of respiratory alkalosis in 28.7–55.4% from other studies that investigated blood gas analysis in hospitalized COVID-19 patients [(5) (55.4%); (6) (28.7%); (7) (30.3%); (8) (40.4%)]. Wu et al. (6) found higher inflammatory markers in respiratory alkalosis group, and other studies corroborate admission hypocapnia as a marker of severe disease (17, 18).

No previous study concurrently assessed ABG, respiratory rate and perception of dyspnoea, and therefore could not directly comment on appropriateness of physiological and perception response, which is a unique aspect here.

Outside the setting of COVID-19, few studies characterize and analyse the ventilatory response in the context of respiratory infection. Although a few studies report prevalence of hypocapnia in community-acquired pneumonia (19–21), none, to our knowledge, report the prevalence of hypocapnia to the extent of alkalosis (PaCO2 <4.6kPa and pH>7.45); therefore, those findings cannot be directly compared.

Profound hypocapnia is found in the context of critical illness, and when prolonged, may adversely influence outcome (15). However, most critically ill patients with abnormal ventilatory responses present with insufficiency (i.e. with acidemia) rather than excessiveness (22). Inappropriate perception of dyspnea is even less well studied. Impaired perception of dypsnoea is more often found in patients with a history of near fatal asthma (23–25), and is associated with impaired chemosensitivity (23, 26) and downregulation of insular activity (27).

We also explored the potential processes that drive excessive tachypnoea in the inappropriate responders group, as well as the impaired perception of dyspnoea to these breathing patterns, and how these acute findings relate to post-covid syndrome.

### What Drives Excessive Tachypnoea?

Breathing is controlled by various physiological mechanisms. At a systems level, hypercapnia (increase in PaCO_2_) and acidosis are the principal chemical drivers of spontaneous automatic breathing, while hypoxia drives breathing only at severe hypoxemia (i.e. PaO_2_ < 6.6 kPa). In addition, thermal, peripheral pulmonary afferents, sympathetic, emotional and somatosensory drives provide adaptive value for particular situations. When awake, further signals provide wakefulness-related drive to breath which underlies why hypocapnia during wakefulness, but not sleep or anesthesia, does not cause apnoea (28). At the highest level, we can voluntarily control breathing through top-down influence of lower breathing centers.

**Figure 3:**
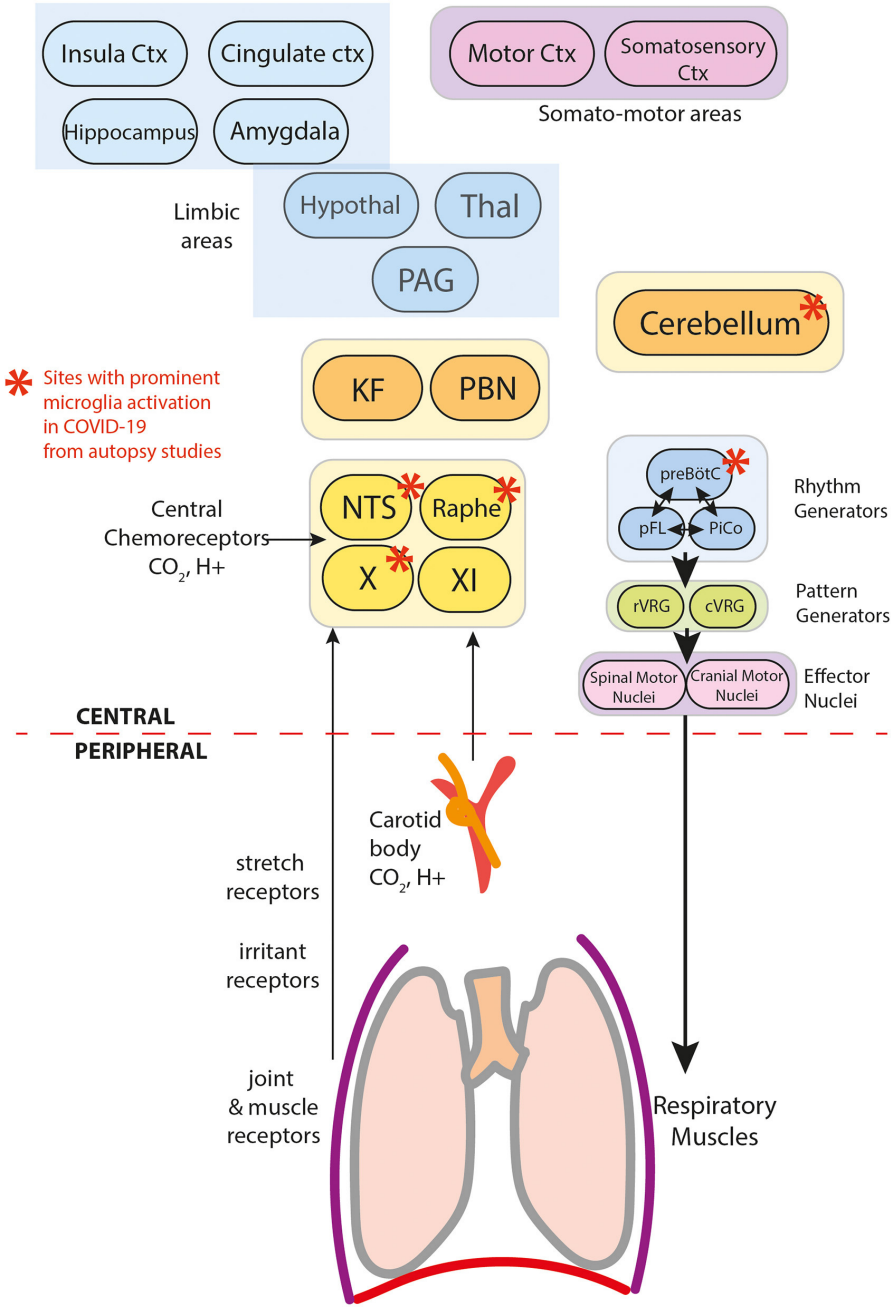
Components of breathing control in the context of COVID-19.

At a biological level, these physiological mechanisms span all levels of the neuraxis from the periphery to central areas illustrated in Figure 3. These drivers interact in a complex non-linear fashion across large-scale neural networks that control breathing. The final common downstream pathway includes central rhythm generators (pre-Botzinger, parafacial respiratory group, and post-inspiratory complex) that output to the central pattern generators (rostral and caudal ventral respiratory group) and then onwards to spinal and cranial motor nuclei and their neuromuscular efferent arm (29).

The effects of COVID-19 on breathing control can occur at multiple levels of regulatory control. Here, we explore evidence of COVID-19 effects at various planes, and document disruption inferred from these data.

#### Hypoxic Drive

Severe hypoxemia (i.e. PaO_2_ < 6.6kPa) drives breathing through the hypoxic ventilatory reflex (HVR) (9–11). In the periphery, arterial chemoreceptors located in the carotid bodies (CB) (Figure 3) sense PaO_2_. Aortic bodies play a minimal role, except when carotid bodies are impaired (ref). Oxygen-regulating cells are also present centrally in the caudal hypothalamus, posterior thalamus, periaqueductal gray, nucleus tactus solitarii (NTS) and the rostral ventrolateral medulla (RVLM)(ref). The CB provide the dominant drive for the HVR, since CB denervation significantly attenuates the response (ref). Central oxygen sensors play a role in severe hypoxia-induced tachypnoea in animal models of carotid-deafferented animals (30).

Given that O_2_-sensing glomus cells express ACE2 (a SARS-CoV-2 receptor), direct infection and impairment of the carotid body could either abolish the HVR or cause abnormal excitability resulting in excessive CB-driven HVR.

However, our results do not support that scenario, since very few patients (5%) had sufficient hypoxemia to drive ventilation. Additionally, hypocapnia observed in our patients would further blunt the HVR.

We cannot exclude pre-hospital periods of sustained severe hypoxia, which could sensitize the CB. Such sensitization results in hyperventilation and increased sympathetic activity that is sustained even after reversal of sustained hypoxic insult, and slowly declines over a few days (31). That more patients in the inappropriate responder group required supplemental oxygen on admission, suggests that this group had higher rates of pre-hospital hypoxia which could sensitize the CB.

#### CO_2_ and pH Homeostasis

Central chemoreceptors that sense PaCO_2_ and pH, are found in the brainstem, cerebellum, hypothalamus and midbrain (32). Those sensors monitor brain interstitial pH, which reflects the integration of PaCO_2_, cerebral blood flow (CBF), and cerebral metabolic rate. CBF itself responds to changes in PaCO_2_ (cerebral autoregulation). A set-point exists which keeps PaCO_2_ and pH in a relatively narrow range. Hypercapnia (PaCO_2_>4.6kPa) or acidosis (pH <7.35) drives hyperventilation. Conversely, hypocapnia (PaCO_2_ <4.6kPa) or alkalosis (pH >7.45) drives hypoventilation. The inappropriate responders group showed hypocapnia *and* alkalosis, which should cause hypoventilation (RR <12), yet they paradoxically are hyperventilating (RR>20). This disturbance in normal homeostasis requires explanation.

PCR-positive SARS-CoV-2 is present at autopsy in the brainstem and cerebellum, specifically in vascular and glial cells, but not neurons, along with activated microglia and evidence of secondary neuronal damage in chemosensitive areas. Specific affected areas include CN X, NTS, dorsal raphe nuclei and cerebellum (13, 33–36) (Figure 3). As such, dysfunction in this redundant network of chemoreceptors appears plausible. Serotonergic neurons of the dorsal raphe, with their extensive projections to motor and respiratory regulatory areas, especially to the cerebellum, are of particular concern.

**Figure 4:**
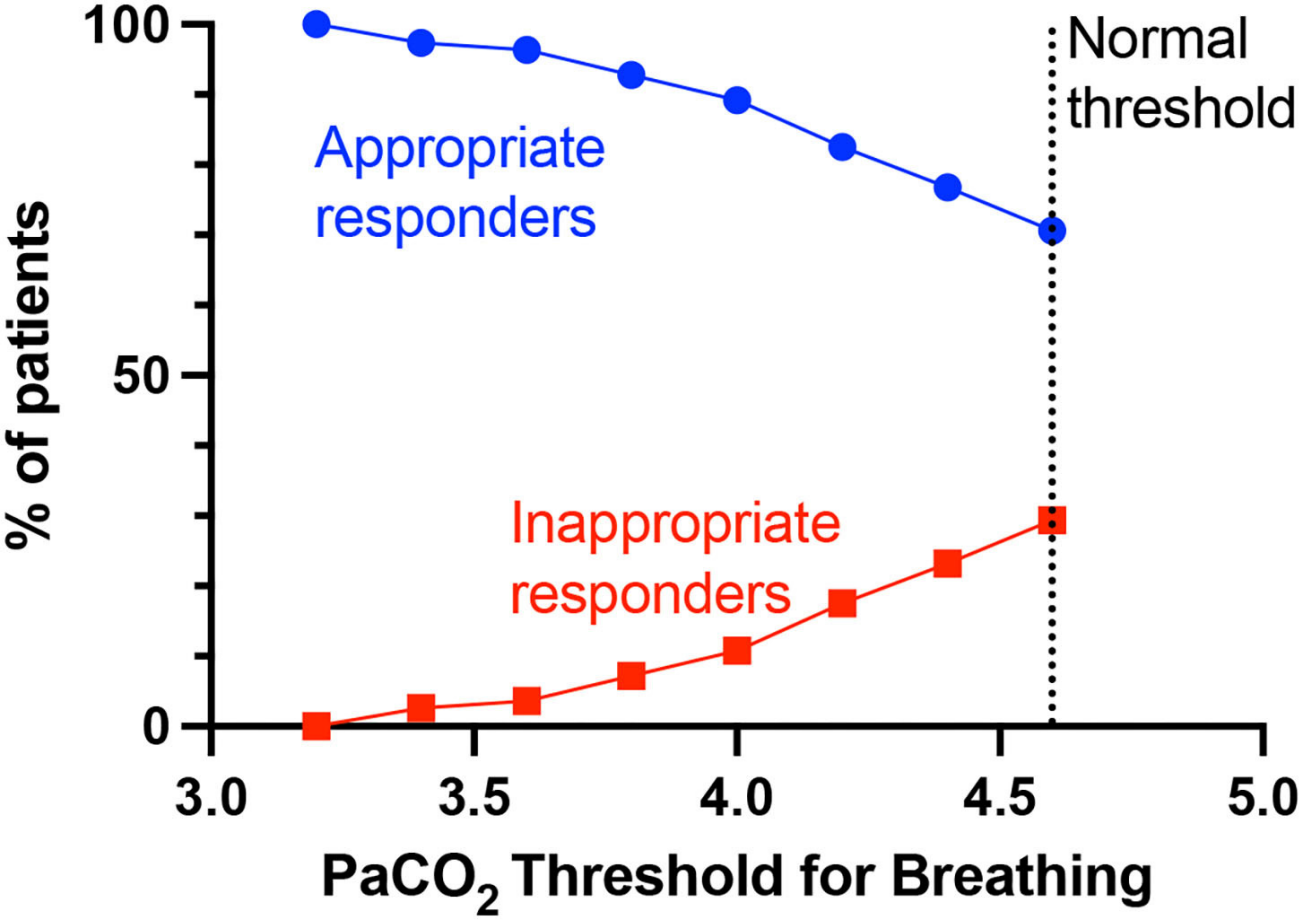
PaCO_2_ thresholds for breathing and % appropriate and inappropriate responders. The normal PaCO_2_ threshold for breathing is >4.6 kPa, below which PaCO_2_ as a breathing drive would be suppressed. Lowering the PaCO_2_ threshold (set-point) for breathing, will decrease the proportion of patients who are considered inappropriate responders for that particular PaCO_2_ threshold i.e., still simultaneously have tachypnoea (RR>20) and alkalosis (pH>7.45).

Two possible mechanisms are conceivable:

First is rheostasis – i.e., shifting the setpoint lower such that hyperventilation is driven by lower PaCO_2_ than the normal 4.6 kPa. Figure 4 shows that lowering the threshold at which PaCO_2_ drives breathing (i.e., lower than the normal set-point/threshold of 4.6 kPa), lowers the proportion of inappropriate responders i.e., those who still have simultaneous tachypnoea (RR>20) and alkalosis (pH>7.45) at the new PaCO_2_ threshold. Rheostasis is normally an adaptive process in homeostatic systems to a sustained change in the environment, such as increased core temperature setpoint during infections, or vestibular-ocular reflex set-points after prolonged stimulation or imbalance in vestibular input (37). Supportive evidence for this hypothesis predicts a rebound hypoventilation when the setpoint returns to normal after the acute insult is removed; that possibility has not been tested in COVID-19 patients.

A second potential mechanism is added bias/additional ventilatory drives.

#### Aberrant Peripheral Sensing

Peripheral drivers via pulmonary vagal C-fibers and slow-adapting mechanoreceptors (SARS) provide sensory feedback to central respiratory centers on local chemical and mechanical conditions. Pulmonary vagal C-fibers fibers are sensitive to inflammatory mediators (including histamine, bradykinin, and prostaglandins), and are consistently activated in lung oedema (38) and experimental acute lung injury (39). These C-fibers can modulate ventilation (increase RR and decrease tidal volume) (40), possibly through vagally-mediated cytokine release in the brainstem (41).

Slow-adapting mechanoreceptors are normally activated by lung inflation, and inhibit central chemoreception (42). Peripheral drives from these sensors may explain hyperventilation in pulmonary oedema, pulmonary fibrosis and pulmonary embolism which persists in the absence of hypercapnia or severe hypoxemia (43).

Our data support a role for these peripheral receptors. This inference is based on the observation of a higher prevalence of more-severe lung disease in the hypocapnic group. Acute respiratory distress syndrome increases RR before impairing gas exchange in rodent models, suggesting an initial role for peripheral afferent stimulation. The acute lung inflammation found in COVID-19 would be expected to stimulate SARs in a similar manner.

#### Other Breathing Drives

##### Thermal Drive

Animals can regulate their body temperature with an increase of core temperature by 1°C, triggering hyperventilation to induce heat loss (44). Our data, showing a correlation between temperature and RR, support this relationship. The significantly higher temperature in the inappropriate responder group suggests a contribution of thermal drive to tachypnoea.

##### Diminished “Higher” Drivers of Breathing

Normally, higher brain centers influence breathing to allow flexible control of breathing with emotion, experience and context, and provide signals involved in the “wakefulness drive to breathe” (28). Our study assessed measures of breathing throughout the day and night. A remarkable finding was that little change in tachypnoea was found in sleeping periods. Although classification of sleep states was unavailable, the data indicate that the normal slowing of respiratory rates with quiet sleep did not occur. That finding is significant, since it points to an abolition of the descending brain influences that mediate control of breathing during sleep states. Although descending limbic and thalamic drives, such as airflow, olfactory or temperature influences may be exerting timing effects, the timing influences that normally slow breathing during sleep appear to be ineffective.

#### Systemic Inflammation

Systemic inflammation itself increases respiratory drive. Our data support greater hyperinflammation in the inappropriate responder group. Hypocapnia is seen in critically ill patients, systemic inflammatory response syndrome, and liver failure (45). In COVID-19, hyperinflammatory responses contribute to disease severity and mortality (46).

#### Central Neurogenic Hyperventilation

In the inappropriate responders group, we found a higher prevalence of anosmia (21 vs. 15%), dysgeusia (25 vs. 12%), headache (33 vs. 23%) and nausea (33 vs. 14%) with similar rates of new anxiety/depression (26 vs. 23%), but a lower incidence of past neurological or psychiatric diagnoses (5 vs. 21%) compared to appropriate responders. These findings warrant exploration of possible central neurogenic contributions to hyperventilation.

The demonstration of COVID-19 influences on the olfactory apparatus (36) and the role of those structures on sensing CO_2_ and other aspects of air passage, as well as the known injury to the amygdala and other limbic structures mediating taste and drive to respiratory phase switching areas of the parabrachial pons (47) and thus, respiratory rate, provide a number of potential central mechanisms to mediate the findings here. Central neurogenic hyperventilation has been reported in other conditions involving immune dysfunction, including multiple sclerosis (48), anti-NMDA receptor encephalitis (49), neuro-Behcet's (50) and Bickerstaff encephalitis (51). Interestingly, marked tachypnoea was the predominant respiratory phenotype of the 1918–1925 epidemic of encephalitis lethargica (52), a disease with recent evidence suggestive of an immune-mediated pathogenesis (53).

Anosmia and dysgeusia are the most prevalent neurological symptoms in COVID-19, suggesting key roles for forebrain limbic structures (Figure 3), particularly olfactory and amygdala structures. However, the weight of evidence supports an interpretation that anosmia results predominantly from SARS-CoV-2 infection of non-neuronal cells in the olfactory epithelium and olfactory bulb (54), and dysgeusia more likely results from peripheral damage to ACE-2-expressing cells of taste buds and peripheral chemoreceptors, or cranial nerves responsible for gustation (CN VII, IX, or X) (55).

Centrally, olfaction is processed by multiple cortical and subcortical regions (56), in particular temporal lobe areas, including the piriform and entorhinal cortex, hippocampus, parahippocampus, amygdala and extra-temporal areas such as the orbitofrontal cortex. Amongst these structures, the hippocampus and amygdala are critical subcortical structures controlling breathing (57, 58).

Central gustatory areas include the NTS, parabrachial nucleus, gustatory thalamus (ventropostero-medial nucleus), amygdala basolateral nucleus and central nucleus, insula cortex, orbitofrontal cortex and anterior cingulate cortex. Among these structures, the NTS and parabrachial nuclei are chemosensitive brainstem structures or receive afferent signals mediating control of breathing. The higher prevalence of nausea in the inappropriate responder group supports involvement of the NTS and parabrachial nuclei. Of interest, the nausea finding is corroborated by the significantly higher prevalence of vomiting in the respiratory alkalosis group (21.2%) compared to the non-respiratory alkalosis group (7.3%) in a previous study (6).

Headache as a symptom has no specific localization, but hints at involvement of CN V (trigeminal nuclei). Another study of hospitalized COVID-19 patients identified the presence of new-onset headache in those presenting without dyspnoea, who also presented earlier (4). This finding raises the possibility of early activation of the trigeminal-vascular system, a concept supported by neuropathological studies showing neuroinvasive potential of SARS-CoV-2 to the brainstem (36). CN V plays a major role in respiratory timing through airflow receptors in the nasal and oral cavities and motor activation of the upper airway musculature (59). These timing roles are especially important for preventing obstructive apnea and maintaining appropriate coordination of cerebellar and pontine respiratory timing circuitry through airflow and thermal afferent activity to the parabrachial pons, a major site of respiratory phase switching (and thus, respiratory timing). The thermal role can be readily demonstrated through cold water facial immersion, which results in immediate apnea, while warming results in tachypnoea and panting.

The amygdala, insula and anterior cingulate cortex, all injured in COVID-19, also serve critical respiratory roles, integrating afferent input from a wide range of receptors and sending projections to other amygdala structures and the hypothalamus; the central nucleus of the amygdala has prominent projections to the parabrachial pons and can influence respiratory rate, even to the point of pacing inspiratory efforts (60). The hypothalamus provides substantial thermal drive to breathing, perhaps influencing the significant role we found for breathing rate and temperature.

Autopsy reports in COVID-19 indicate local immune-mediated activity in the brainstem and cerebellum (13). The cerebellum plays a critical role in respiratory timing, coordinating afferent stimuli from multiple somatic and vascular sites and essential timing circuitry with the parabrachial pons. The cerebellar fastigial nuclei are particularly important in these ventilatory roles, specifically during chemical stress and not during eupnoea. Injury to the fastigial nuclei, such as in Central Congenital Hypoventilation Syndrome or heart failure patients, distorts both amplitude and timing to ventilatory and blood pressure challenges (61, 62).

#### Sensitization of the Efferent Arm

A possible source of hyperventilation lies in the efferent arm. There is no evidence to suggest dysfunction in the motor nuclei, motor neurons or muscles. Nevertheless, pre-admission sustained hypoxia could centrally sensitize motor neurons driving the phrenic nerves, enhancing phrenic output. However, the principal findings suggest a timing dysfunction, i.e., a rate, not motor effort, issue.

Overall, the data presented here suggest that tachypnoea was driven by both peripheral and central mechanisms, but not hypoxia.

### What Drives Impaired Perception of Dyspnoea to Tachypnoea?

Increased afferent feedback from chest wall mechanoreceptors and muscle stretch receptors with increased RR is usually perceived as breathlessness (63). It is abnormal that over a third of patients in the inappropriate responder group had reduced dyspnoeic response to tachypnoea.

First, neuromechanical coupling may be maintained in this COVID-19 cohort due to relatively preserved lung compliance (64). This coupling is unusual for most disorders that lead to acute lung injury. To a large degree, this interpretation explains the lack of dyspnoea, because the mechanoreceptor activity should continue to be proportional to the predicted activity from a given motor signal that drives ventilation. Therefore, there should not be an error signal, which should indicate no dyspnoea. This possibility has been supported by others (64, 65).

Secondly, interruptions in central processes that compare expected consequences of (breathing) motor commands and the actual consequences (feedback from periphery) may occur. Normally the “error” signal generated from a mismatch between these expected and actual consequences would generate the dyspnoeic perception of “increased work of breathing” (66). Both the cerebellum and insula play major roles in the perception of dyspnoea (67), as well as their aforementioned roles in control of ventilation. Damage to the cerebellum could impair gain and timing of these signals. Alternatively, a shift in setpoint to a higher threshold for dyspnoea perception would require a higher RR to perceive dyspnoea. Here, one possibility is a downregulation of insula activity. In patients with asthma, downregulation of affect-related insula cortex activity correlates with blunted perception of dyspnoea (68). Lesions in the right insular cortex are associated with blunted dyspnoea (69).

### How Do These Acute Findings Relate to Post-COVID Syndrome in a Subset of Patients?

The prevalence of breathing pattern disorder (BPD) at 6 weeks post-discharge was (24%) and in the absence of other neurological findings, or previous respiratory, neurological, or psychiatric disorder diagnoses. Notably, most patients recovered over time. The pathophysiology of breathing pattern disorder is poorly understood, but involves abnormal breathing rate, pattern and inappropriate dyspnoea. The neural mechanisms underlying the recovery are not understood.

Whether mechanisms of post-Covid breathing pattern disorder can be inferred from our data is unclear. Only 62.5% of patients who had BPD were inappropriate responders in the acute phase – for this group, rheostasis may be explanatory– a shift in set-point during the acute phase to a higher state. Such a shift is likely followed by a resetting after the acute illness that disturbs breathing perception and results in the high prevalence of breathing disorder found in our cohort. Our data suggest that by 1 year after the acute insult, the set-point has reset to its pre-Covid state.

Future work should focus on prospective cohort studies of hospitalized COVID-19 patients, with an emphasis on gathering more objective respiratory rate using wearable devices, more quantitative measures of perception of dyspnea over multiple intervals from admission to discharge, and prolonged follow-up. Correlation of respiratory patterning with cardiovascular changes would also be useful. Determination of respiratory patterning during the normally short-lasting periods of rapid eye movement sleep would help differentiate whether COVID-19 impacts breathing differently during that state, thus helping to determine abberant influences. Additional functional neuroimaging of subsets of patients with impaired ventilatory and perceptual response would further mechanistic understanding. More broadly, it remains unanswered whether the phenomena we observe here are unique to COVID-19 or are found in other respiratory conditions – further studies are needed.

### Clinical Implications

We show (1) that dyspnoea alone poorly correlates with disease severity or degree of hypoxia, despite its inclusion in many severity triage scoring systems; (2) tachypnoea appears to be a more useful clinical marker, as it is common, and correlated with more severe pulmonary disease; (3) our study supports the use of early blood gas analysis - with hypocapnia and respiratory alkalosis being of particular concern, because this group has more severe disease; (4) we suggest that acute impairment in breathing control may lead to dysfunctional breathing that is prolonged, but will likely resolve by 1 year. The unresponsiveness of control mechanisms to extreme values in pH and oxygenation mandate further studies into processes mediating disruption of sensory, integrative central processing, and motor output on respiration, and the activities underlying recovery of longer-term effects of COVID-19 on breathing control.

### Limitations of the Study

The limitations include a relatively small number of subjects, and that the data are derived from a single center. However, the study is from a geographical location with a highly heterogenous population, providing a wide representation of physiological presentation. The study is also a retrospective design, and therefore, no formal protocolised assessment of dyspnoea was available, nor were comments on hyperpnoea. Not unique to the study is the difficulty of counting RR in clinical situations. However, the persistence of tachypnoea over multiple recordings argues for the validity of the data. Inherently, the perception of dyspnoea is subjective and multi-dimensional – but our inclusion criteria for recording dyspnoea covers these multi-dimensional descriptors. We also had limited detailed data on other autonomic aspects including cardiac patterning. We had no neuropsychometric assessment, for practical reasons during that phase of the COVID-19 pandemic which may have revealed subtler psychological localisable deficits. Although we note that nearly a third of patients in our study had impaired homeostatic control of ventilation, the study only included 194 (who met the inclusion criteria) out of the 492 patients admitted. Therefore, the lowest bound of the prevalence estimate would be 11.6% (57/492). Finally, the number of patients who attended follow-up appointment was low, which limited inferences of post-acute Covid effects.

## Data Availability Statement

The raw data supporting the conclusions of this article will be made available by the authors, without undue reservation.

## Ethics Statement

The studies involving human participants were reviewed and the study was approved by the Westminster Research Ethics Committee (NHS Health Research Authority, IRAS no: 284088). Written informed consent for participation was not required for this study in accordance with the national legislation and the institutional requirements.

## Author Contributions

PJ, CZ, RH, and RA: acquisition and analysis or interpretation of data. PJ, RH, and RA: drafting of the manuscript, administrative, and technical or material support. PJ: statistical analysis. RH and RA: obtained funding and supervision. All authors: concept and design and critical revision of the manuscript for important intellectual content. All authors contributed to the article and approved the submitted version.

## Funding

This research was supported in part by the Fidelity Charitable Nancy Adams and Scott Schoen Fund and the Kraig and Linda Kupiec Family Trust.

## Conflict of Interest

The authors declare that the research was conducted in the absence of any commercial or financial relationships that could be construed as a potential conflict of interest.

## Publisher's Note

All claims expressed in this article are solely those of the authors and do not necessarily represent those of their affiliated organizations, or those of the publisher, the editors and the reviewers. Any product that may be evaluated in this article, or claim that may be made by its manufacturer, is not guaranteed or endorsed by the publisher.
